# Journal Special to the U.S.V.M.A.—Omaha, September 6-9, 1898

**Published:** 1898-07

**Authors:** 


### JOURNAL SPECIAL TO THE U. S. V. M. A.—OMAHA, SEPTEMBER 6–9, 1898.

As the time is fast approaching for the members of the profession to determine upon their plans for reaching the convention city of our 1898 meeting, it has been decided to, so far as possible, go in a body, and for this end the following plan has been adopted as the one best suited for the largest number of our members both East and West. After considering the merits of the several roads leading from the East to the Missouri River, the one affording the most direct and convenient arrangements will be the Pennsylvania Railroad from New York, Philadelphia, Baltimore, Washington, and Pittsburg to Chicago, and thence via the Burlington route (C., B. & Q.) from Chicago to Omaha.

Our plans are to centre at Chicago from all diverging points as many members of the profession as possible, and should the number warrant it, the Burlington route have agreed to run a *Journal Special* from Chicago to Omaha.

*Itinerary.*—Leave New York September 3d, 2 P.M.; Philadelphia, 4.50 P.M.; Baltimore, 4.35 P.M.; Washington, 3.30 P.M.; Harrisburg, 7.30 P.M.; Pittsburg, 1.00 A.M. Arrive at Chicago, September 4th, 5 P.M. Leave Chicago, September 4th, 5.50 P.M. Arrive Omaha, September 5th, 8.10 A.M.

Rates authorized by the Trunk Line Association is a fare and one-third on the certificate plan. By this arrangement members of the profession and their friends will purchase from their ticket agent, by the route above mentioned, a regular one-way first-class ticket, taking certificate for the same, which will be honored at Omaha on proper indorsement by joint agent for a return ticket at one-third of amount paid on going trip.

For the information of Eastern delegates, we give below the one-way rates from prominent points to Omaha: New York City, $32.75; Trenton, N. J., $31.80; Philadelphia, $31.00; Wilmington, Del., $31.00; Baltimore, Md., $29.50; Washington, D. C., $29.50; Lancaster, Pa., $30.33; Harrisburg, $29.25. Rates from other points not named can be secured upon application to any ticket agent of the Pennsylvania or connecting roads. Parties residing off the main line of travel can join the special party at points above named en route.

Sleeping-car rates from New York and Philadelphia to Omaha for double berth is $7.50; this, of course, can be reduced one-half where two occupy the same berth. Rates from other points proportionate. As sleeping-car space will have to be paid for when reservations are made, I would request that the above amount accompany order for such reservation. Preference as to location of berths will be given those who apply first.

The entertainment already assured by our colleagues at Omaha will be of such a character that must add in great measure to the attraction and pleasure of this annual professional pilgrimage, and the greater the attendance of the members of the profession, their wives, and those interested in our work, must add much to the fullest enjoyment of this trip.

Those members residing in the South who will not find it convenient to travel via Chicago can use the Burlington route's fast-train service from St. Louis to Omaha, addressing Mr. J. G. Delaplaine, city passenger agent of that line, corner Broadway and Olive Street, St. Louis, Mo., for sleeping-car space.

Those members residing in Canada, northern New York, Michigan, Wisconsin, Ohio, Indiana, and Illinois, joining the *Journal Special* at Chicago can make their reservations by addressing Mr. Frank E. Bell, city passenger agent, Burlington route, 211 S. Clark Street, Chicago, Ill. The rate for double berth from Chicago to Omaha is $2.50.

Those from the East joining the special party may have space reserved for them from New York, Philadelphia, or points named, or from Chicago, by remitting amount to the undersigned.

For further information, address W. HORACE HOSKINS,

Managing Editor of Journal of Comparative Medicine and Veterinary Archives,
3452 Ludlow Street, Philadelphia, Pa.

